# Reviewers are fundamental to success of the International Brazilian Journal of Urology

**DOI:** 10.1590/S1677-5538.IBJU.2024.01.02

**Published:** 2024-03-18

**Affiliations:** 1 Universidade do Estado do Rio de Janeiro Unidade de Pesquisa Urogenital Rio de Janeiro RJ Brasil Unidade de Pesquisa Urogenital - Universidade do Estado do Rio de Janeiro - Uerj, Rio de Janeiro, RJ, Brasil; 2 Hospital Federal da Lagoa Rio de Janeiro RJ Brasil Serviço de Urologia, Hospital Federal da Lagoa, Rio de Janeiro, RJ, Brasil

In 2023 the International Brazilian Journal of Urology received the highest impact factor of his history, and this fact was possible because the serious peer review process of our Journal (1). In this year we received more than 600 papers. The peer review system is a very difficult process because is completely free. This process depends of the hard work of the experts in several topics reviewers by on the topic (2). The Editor-in-Chief would like to thank all the reviewers and specially to the Doctors: **Alexandre Danilovic** (Hospital das Clínicas da Faculdade de Medicina da USP -São Paulo, SP, Brasil); **Rodrigo de Castro** (Universidade Federal de São Paulo - UNIFESP, São Paulo, SP) and **Lars Schimmoeller** (University Hospital Düsseldorf), Germany who reviewed more than 3 articles during the year and strictly within the deadline.

Thanks a lot!!!!!
